# Near-infrared fluorescence angiography for intra-operative assessment of pedicled omentoplasty for filling of a pelvic cavity: a pilot study

**DOI:** 10.1007/s10151-019-02048-0

**Published:** 2019-08-21

**Authors:** M. D. Slooter, R. D. Blok, D. D. Wisselink, C. J. Buskens, W. A. Bemelman, P. J. Tanis, R. Hompes

**Affiliations:** 1 Department of Surgery, Amsterdam UMC, University of Amsterdam, Meibergdreef 9, 1105 AZ Amsterdam, The Netherlands; 2 LEXOR, Center for Experimental and Molecular Medicine, Oncode Institute, Cancer Center Amsterdam, Amsterdam UMC, University of Amsterdam, Meibergdreef 9, 1105 AZ Amsterdam, The Netherlands; 3 Department of Surgery, Amsterdam UMC, University of Amsterdam, G4, Post box 22660, 1100 DD Amsterdam, The Netherlands

**Keywords:** Omentoplasty, Fluorescence angiography, Indocyanine green (ICG), Rectal surgery, Pelvic filling

## Abstract

**Background:**

During creation of a pedicled omentoplasty, blood flow to segments of the omentum might become compromised. If unrecognized, this can lead to omental necrosis. The purpose of this study was to investigate the potential added intra-operative value of the use of fluorescence angiography (FA) with indocyanine green (ICG) to assess omental perfusion.

**Methods:**

All consecutive patients undergoing a pedicled omentoplasty in a 6-month period (April 1 2018–October 1 2018) in a University hospital were included. The primary outcome was change in management due to FA. Secondary outcomes included the amount of additionally resected omentum, added surgical time, and quantitative fluorescent values (time to fluorescent enhancement, contrast quantification).

**Results:**

Fifteen patients had pelvic surgery with omentoplasty and FA. Change in management occurred in 12 patients (80%) and consisted of resecting a median of 44 g (range 12–198 g) of poorly perfused omental areas that were not visible by conventional white light. The median added surgical time for the use of FA and subsequent management was 8 min (range 3–39 min). The first fluorescent signal in the omental tissue appeared after a median of 20 s (range 9–37 s) after injection of ICG. The median signal-to-baseline ratio was 23.7 (interquartile range 12.2–29.7) in well perfused and 2.5 (interquartile range 1.7–4.0) in poorly perfused tissue.

**Conclusions:**

FA of a pedicled omentoplasty allows a real-time assessment of omental perfusion and leads to change in management in 80% of the cases in this pilot study. These findings support the conduct of larger studies to determine the impact on patient outcome in this setting.

## Introduction

Application of omentoplasty is a well-established option for filling of a presacral cavity after both primary abdominoperineal resection [1] and more complex salvage surgery [2–5]. Success of an omentoplasty depends on the adequacy of filling the pelvic cavity, determined by the omental volume and length. After full detachment from the transverse colon, additional mobilization is often necessary. This can be achieved by creation of a pedicled omentoplasty on one of the gastroepiploic arteries. Adequate vascularization of the omental flap is guaranteed as the main trunks are connected via several collateral arches. Nevertheless, intra-operative partial omental flap necrosis has been reported to occur in up to 10% of cases [6], and post-operative flap necrosis requiring reoperation has been reported in an additional 2–4% [6–8]. This suggests that blood flow to segments of the omentum might become compromised after flap creation, implying the need for intra-operative evaluation of omental perfusion with higher sensitivity than conventional white light assessment.

Fluorescence angiography (FA) using indocyanine green (ICG) has emerged as a promising technique for real-time intra-operative evaluation of perfusion [9]. After intravenous administration, ICG rapidly binds to plasma proteins and is transported with minimal leakage to the interstitium, making ICG an ideal marker for perfusion. FA using ICG of the omentum has been described during a free omental lymphatic transplant [10] and a pedicled omental flap to wrap the cervical esophagogastric anastomosis [11]. However, the possible added value of FA for assessing omental perfusion of a pedicled omentoplasty for pelvic filling has not been evaluated so far.

Therefore, the aim of this single-center pilot study was to investigate the potential intra-operative value of the use of FA in patients who underwent omentoplasty for filling of a pelvic cavity.

## Materials and methods

### Patient selection

The Amsterdam University Medical Center (UMC) is a tertiary referral center for the treatment of complex pelvic sequelae of inflammatory bowel disease and treatment of pelvic malignancies. For this reason, a relatively high number of salvage procedures with (pedicled) omentoplasty are performed each year [3, 4]. All consecutive patients having a pedicled omentoplasty in a 6-month period from introduction of FA in April 1 2018 to October 1 2018, in the Amsterdam UMC, were prospectively recorded. The complex pelvic procedures were performed by three specialized colorectal surgeons.

Retrospective data extraction from electronic patient files included demographics, significant past medical history, operative details, and FA video files. Co-morbidities recorded were diabetes, pulmonary, and cardiovascular diseases.

The local ethics committee waived the need for written informed consent, because there was no burden for the patient and data were handled anonymously. Patients were informed about the study by letter, including an opt out form with stamped return envelope. Patients were given the opportunity to opt out within 4 weeks, after which consent to participation was assumed.

### Outcome measures

The primary outcome was change in management due to FA. Secondary outcomes included quality of the technique, additionally excised omental fat (in grams), added surgical time (rounded to minutes), and quantitative fluorescent values (i.e., time to fluorescent enhancement and contrast quantification). Quality of the technique was deemed high if no technical failures occurred and the fluorescent signal was evident for decision making. Other outcomes included feasibility of the technique and safety profile (adverse events related to ICG).

### Procedure

After creation of the pedicled omentoplasty, the perfusion was assessed by visual inspection, with the addition of palpation of feeding vessels in open procedures. Based on these findings, it was decided whether or not additional trimming was necessary. FA was then performed after injection of ICG (0.1 mg/kg/bolus) and time of injection was noted in the electronic patient file. FA was subjectively analyzed by the surgeon. If FA was inconsistent with the initial intended strategy and additional resection was deemed necessary, this was considered change in management. Subsequently, areas of the omentum with poor perfusion were resected accordingly. Imaging was performed by the PINPOINT Endoscopic Fluorescence Imaging System or the SPY Portable Handheld Imaging System (Stryker, Kalamazoo, MI, USA), Food and Drug Administration (FDA)—approved and European Conformity (CE)-marked systems for visualizing blood flow in (micro-)vessels, tissue, and organ perfusion during surgical procedures. The video file of omental FA was not routinely recorded within the patient file.

### Contrast quantification

Images were subtracted from the video files. Signal-to-baseline ratios (SBR) of well perfused and poorly perfused tissue, based on subjective judgement, were calculated. The signal was assessed 30 s after fluorescent enhancement in the target tissue, and the baseline before enhancement. The SBR were measured using the ImageJ software (v 1.50i, National Institutes of Health, Bethesda,MD, USA). Contrast-to-noise ratios (CNR) were calculated according to Tichauer et al. [12].

### Statistical analysis

All categorical data are presented as number of cases and percentages, while continuous data are shown as either median and total range or interquartile range (IQR). To compare perfused to poorly perfused tissue, analysis of significance was performed through paired *t* tests and *p* < 0.05 was considered significant. Data were analyzed using the Statistical Package for Social Sciences (SPSS) of IBM Statistics, version 25.0.

## Results

### Baseline characteristics

Fifteen patients were included in this study. The median age of the patients was 55 years (range 23–82 years) with a median body mass index of 27 kg/m^2^ (range 20–42 kg/m^2^) (Table 1). Six patients had surgery for severe medical refractory fistulizing anorectal Crohn’s disease and nine for sequelae from previous treatment of a pelvic malignancy. Details of the surgical procedures are provided in Table 1. The omentoplasty was pedicled on the left gastroepiploic artery in 9 out of 15 (60%) and on the right in 6 out of 15 (40%) cases. Transmesocolic was the preferred route to the pelvis in most cases.

**Table 1:** Baseline characteristics and surgical procedures

	FA (*N* = 15)
Age, years	55 (range 23–82)
BMI, kg/m^2^	27 (range 20–42)
Comorbidity	
Diabetes mellitus	3/15 (20%)
Pulmonary diseases	1/15 (6.7%)
Cardiovascular diseases	6/15 (40%)
Smoking	
Yes	3/15 (20%)
No	12/15 (80%)
ASA	
< 3	8/15 (53.3%)
3	7/15 (46.7%)
Primary disease	
Crohn’s disease	6/15 (40%)
Rectal cancer	8/15 (53.3%)
Cervical cancer	1/15 (6.7%)
Prior treatment	
Biologicals < 3 months^a^	2/6 (33.3%)
Neoadjuvant (chemo)radiation^b^	7/9 (77.8%)
Prior interventions	
Abscess drainage	4/15 (26.7%)
Ileo-/colostomy	15/15 (100%)
LAR/Hartmann	6/15 (40%)
APR	1/15 (6.7%)
Re-do	1/15 (6.7%)
EVAC	3/15 (20%)
Indication surgery	
Therapy refractory disease	6/6 (100%)
Presacral sinus	6/9 (66.7%)
Other^c^	3/9 (33.3%)
Surgical procedure	
Intersphincteric proct(ocolec)tomy	7/15 (46.7%)
Resection anastomosis	5/15 (33.3%)
Debridement sinus	7/15 (46.7%)
Total exenteration	1/15 (6.7%)
Other	1/15 (6.7%)
Abdominal approach	
Laparoscopy	12/15 (80%)
Laparotomy	3/15 (20%)
Perineal approach	
TAMIS	14/15 (93.3%)
Open	1/15 (6.7%)
Pedicled omentoplasty	
Left gastroepiploic artery	9/15 (60%)
Right gastroepiploic artery	6/15 (40%)
Route to pelvic	
Left paracolic gutter	6/15 (40%)
Trans mesocolic, medial to colostomy	8/15 (53.3%)
Supracolic, medial to colostomy	1/15 (6.7%)

*BMI* Body mass index, *ASA* American Society of Anesthesiologists Score, *LAR* Low anterior resection, *APR* Abdominoperineal resection, *EVAC* Endoscopic vacuum-assisted closure, *TAMIS* Transanal minimally invasive surgery

^a^The patients with Crohn’s disease

^b^The patients with malign diseases (rectal and cervical cancer)

^c^Namely: radiation necrosis with vesico- and rectovaginal fistulas, near circumferential anastomotic dehiscence with pelvic sepsis, or synchronous double tumor of colon/rectum, blow-out colon and abscess

### Fluorescence angiography

**Fig. 1:**
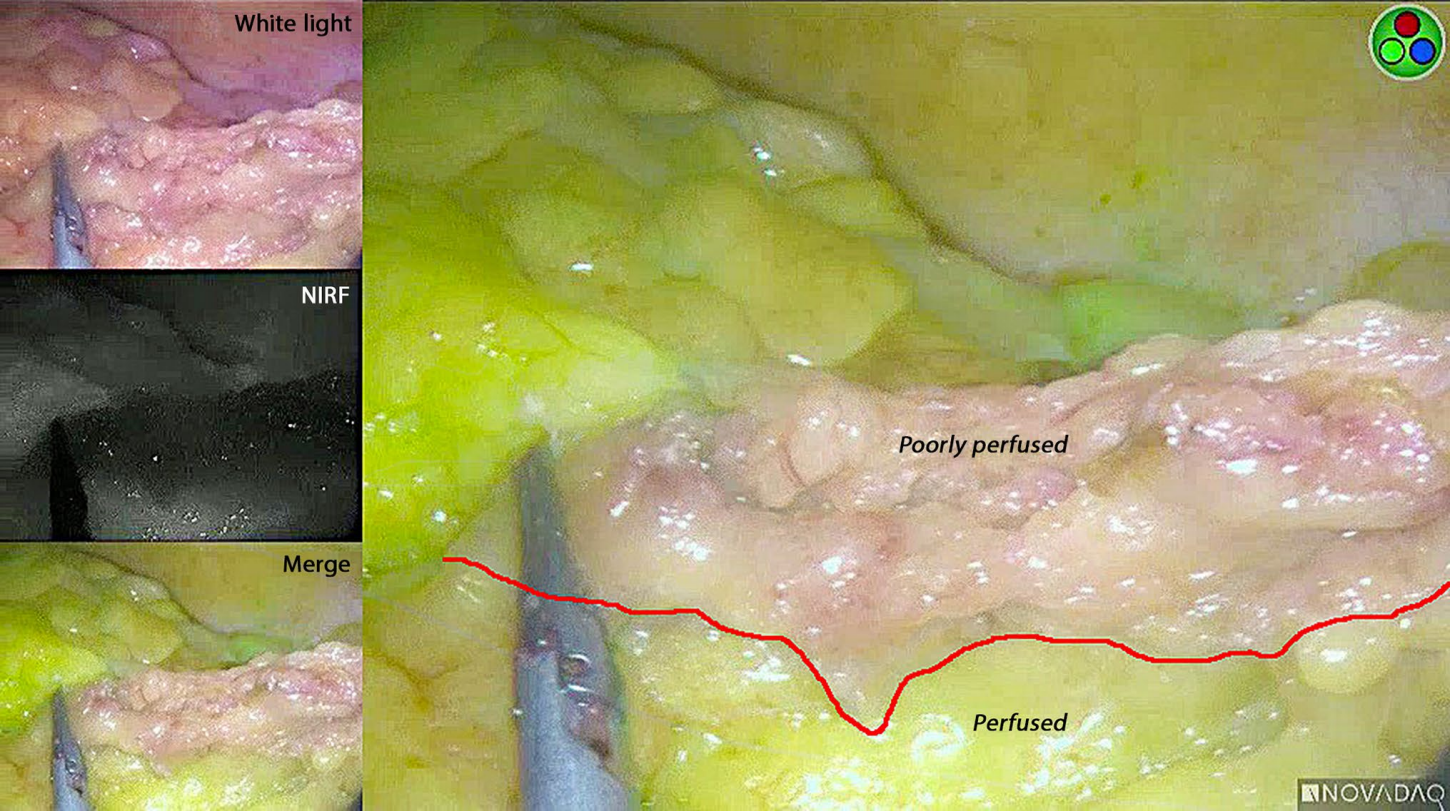
An example of fluorescence angiography after a pedicled omental flap creation. White light is the standard view and shows no areas suspect for decreased perfusion. NIRF is the near-infrared fluorescent signal of ICG. Merge is the overlay of the white light and NIRF, with the fluorescent signal displayed in pseudo green. The technique points out the areas that are perfused and poorly perfused. The red line demonstrates the demarcation line along which additional tissue was excised

The use of FA was performed at least 60 min after the creation of the omentoplasty for optimal conventional white light assessment. A high-quality intra-operative ICG angiogram was achieved in all patients. Figure 1 shows an example of FA after creation of the omental pedicle. The use of FA led to a change in management in 12 out of 15 patients (80%) (Table 2). In 12 patients, poorly perfused omental fat was excised on the adjusted demarcation line, removing a median amount of 44 g (range 12–198 g). For 11 procedures (4 video clips were not recorded), the median added surgical time was 8 min (range 3–39 min), including time for perfusion assessment and trimming of the omental flap. No adverse events related to ICG occurred.

**Table 2:** Fluorescence angiography

	FA (*N* = 15)
Change in management	12/15 (80%)
Additional excised tissue (g)	44 (12–198)
Added surgical time (min)	8 (3–39)
Time of injection to first fluorescence (s)	20 (9–37)
Time of first fluorescence to demarcation line (s)	12 (7–22)

### Contrast quantification

In 10 procedures, the first fluorescent signal in the omental tissue appeared at a median 20 s (range 9–37 s) after injection of ICG. After first fluorescent enhancement, the demarcation line was clearly visible after median 12 s (range 7–22 s).

**Fig. 2:**
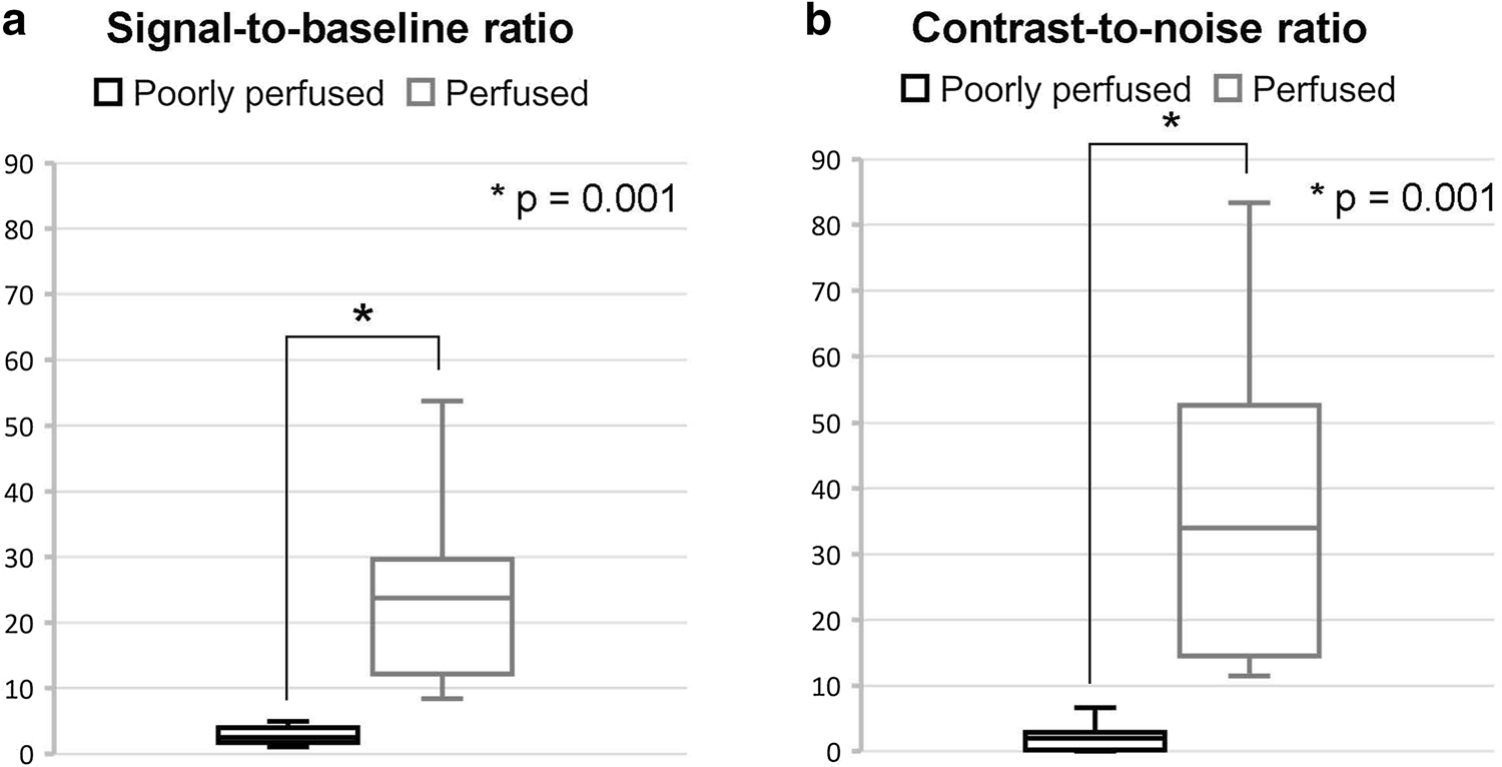
Contrast quantification **a** signal-to-baseline ratio, **b** contrast-to-noise ratio

In 11 procedures, the difference between the SBR and CNR of perfused versus poorly perfused tissue significantly differed (Fig. 2). The median SBR was 23.7 (IQR 12.2–29.7) in well-perfused tissue and 2.5 (IQR 1.7–4.0) in poorly perfused tissue (Fig. 2a). Corresponding CNR was 34.0 (IQR 14.5–52.7) and 2.2 (IQR 0.7–3.2), respectively (Fig. 2b).

## Discussion

This study illustrates the potential added intra-operative value of FA to assess omental perfusion after creation of a pedicled omentoplasty. In this study, we found a 80% change in management due to FA.

The high rate of change in management due to devascularization is quite remarkable, as surgeons judge omental perfusion to be insufficient in approximately 10% of cases by means of conventional white light assessment [6]. This is also contradictory to the low incidence of post-operative omental necrosis requiring reoperation as reported in the current literature [6–8]. However, there is probably an important underreporting of necrosis as literature on omentoplasty solely concerns retrospective studies and describes incidental cases of omental necrosis that resulted in surgical reintervention. Small pieces of omentum that become ischemic are potentially overlooked or misdiagnosed. An ischemic tip of the omentum might not lead to apparent clinical deterioration. But more likely, this leads to a pelvic fluid collection that is interpreted as a consequence of the underlying condition. If such an abscess is radiologically drained, the etiology is hard to determine.

Another explanation for the high rate of change in management is overtreatment: additional tissue might be resected without clinical relevance. Necrosis of small parts of omentum might not always lead to an abscess or wound healing problems. However, it is important to note that necrosis of small parts of omentum is more likely to lead to infectious problems in patients suffering from pelvic sepsis, in comparison to a primary clean-contaminated abdominoperineal resection for rectal cancer.

Another reason for the high rate of change in management in this study could be related to the inadequate threshold of detection of ICG. Although contrast quantification showed a clear difference between the subjectively judged perfused and poorly perfused tissue, the ratios found in the group with poorly perfused tissue remain above the cutoff values described in the literature (a SBR above two is considered clinically and a CNR above three as statistically fluorescence enhanced [13]). This raises the question if the poorly perfused tissue might be adequately perfused, while subjective interpretation determines that the fluorescent dye is not visible. Currently the threshold of the amount of dye or signal for adequate perfusion of a pedicled omentoplasty is unknown. Further work is required to determine optimum sensitivity and threshold levels for the assessment of perfusion sufficiency, in particular with regard to omentum-related complications. A small number of studies have been performed to determine threshold levels by quantification of FA in animal and human studies, with promising findings that might guide future FA [14–16].

This study was limited by the relatively low number of patients and absence of patient outcomes. Therefore, although a high rate of change in management due to FA of a pedicled omentoplasty was found, no definitive conclusions can yet be drawn about its clinical relevance. To prove the potential added value of intra-operative fluorescence angiography, follow-up studies with a control group are warranted to compare clinical patient outcomes.

## Conclusions

This single-center experience using FA to assess perfusion of a pedicled omentoplasty for filling of a pelvic cavity shows that the technique is feasible and readily achievable with minimal added operating time. The high rate of change in management due to FA indicates that larger studies should be conducted to determine the impact on patient outcome in this setting.
